# Performance of the Surgical Pleth Index and Analgesia Nociception Index in Healthy Volunteers and Parturients

**DOI:** 10.3389/fphys.2021.554026

**Published:** 2021-03-08

**Authors:** Byung-Moon Choi, Hangsik Shin, Joo-Hyun Lee, Ji-Yeon Bang, Eun-Kyung Lee, Gyu-Jeong Noh

**Affiliations:** ^1^Department of Anesthesiology and Pain Medicine, Asan Medical Center, University of Ulsan College of Medicine, Seoul, South Korea; ^2^Department of Biomedical Engineering, College of Engineering, Chonnam National University, Yeosu, South Korea; ^3^Department of Anesthesiology and Pain Medicine, International St. Mary’s Hospital, Catholic Kwandong University, Incheon, South Korea; ^4^Department of Statistics, Ewha Womans University, Seoul, South Korea; ^5^Department of Clinical Pharmacology and Therapeutics, Asan Medical Center, University of Ulsan College of Medicine, Seoul, South Korea

**Keywords:** index, pain quantification, volunteers, parturient, physiologic change

## Abstract

Various commercially available nociception devices have been developed to quantify intraoperative pain. The Surgical Pleth Index (SPI) and Analgesia Nociception Index (ANI) are among the analgesic indices that have been widely used for the evaluation of surgical patients. This study aimed to evaluate the clinical performance of the SPI and ANI in conscious healthy volunteers and parturients. Ten healthy volunteers and 10 parturients participated in this study. An algometer was used to induce bone pain in the volunteers until they rated their pain as five on the numerical rating scale (NRS); this procedure was repeated during the administration of remifentanil or normal saline. The study comprised two periods, and the volunteers were infused with different solutions in each period: normal saline during one period and remifentanil during the other in a randomized order. The parturients’ SPI and ANI data were collected for 2 min when they rated their pain levels as 0, 5, and 7 on the NRS, respectively. Both the SPI and ANI values differed significantly between NRS 0 and NRS 5 (*P* < 0.001) in the volunteers, irrespective of the solution administered (remifentanil or normal saline). At NRS 5, the SPI showed similar values, irrespective of remifentanil administration, while the ANI showed significantly lower values on remifentanil administration (*P* = 0.028). The SPI and ANI values at NRS 5 and NRS 7 did not differ significantly in the parturients (*P* = 0.101 for SPI, *P* = 0.687 for ANI). Thus, the SPI and ANI were effective indices for detecting pain in healthy volunteers and parturients.

## Introduction

Various commercially available nociception devices have been developed for the quantification of intraoperative pain (Ledowski, 2019). These devices possess distinct algorithms that detect the changes in the autonomic nervous system in response to surgical stress (Ledowski, 2019). The performance of analgesic indices such as the Surgical Pleth Index (SPI, GE Healthcare, Milwaukee, WI, United States) and Analgesia Nociception Index (ANI; MetroDoloris^TM^, Loos, France) has been widely evaluated mainly in surgical patients (Gruenewald et al., 2015; Ledowski et al., 2016; Chanques et al., 2017). The SPI evaluates peripheral vasoconstriction and cardiac autonomic tone using two variables, i.e., the heartbeat interval (HBI) and photoplethysmographic amplitude (PPGA). The equation for calculating the SPI value is as follows: SPI = 100 − (0.7 × PPGAnorm + 0.3 × HBInorm), where PPGAnorm and HBInorm stand for the normalized PPGA and HBI, respectively (Ledowski, 2019). The value of the SPI can lie between 0 and 100 and approaches zero with the activation of the sympathetic nerves. The ANI calculates the area under the curve of the high-frequency spectrum of heart rate variability (HRV) and presents it as a value ranging from 0 to 100 (Ledowski, 2019). The ANI value approaches 100 as the parasympathetic nerves are activated. The SPI and ANI should reflect the degree of pain as accurately as possible if they are to be used as meaningful quantitative surrogate measures of pain. If the intensity of the pain felt by the patient is the same, irrespective of analgesic administration, analgesic indices that show similar values for measurements performed in conditions with and without analgesic administration will be more useful. This is because pain management is performed according to the pain score in actual clinical practice [e.g., numerical rating scale (NRS)], which is calculated according to the rating provided by the patient. A recent meta-analysis showed that SPI-guided anesthesia reduced opioid consumption (Won et al., 2018). ANI scores >50 had a high negative predictive value for moderate or severe pain (Boselli et al., 2014). However, studies evaluating the performance of these indices in conscious healthy volunteers are scarce (Yan et al., 2017). The advantage of volunteer-based studies is that they permit the application of numerous methods that cannot be easily used in patient-based studies. Therefore, the performance of these indices should be evaluated using well-controlled volunteer studies.

Another useful study design for accurately assessing the clinical performance of nociception indices entails enrolling participants who feel pain over a wide spectrum of intensity within the same environment. Parturients feel different degrees of pain depending on the cycle of uterine contraction (Labor and Maguire, 2008). The neurophysiology of labor pain can be characterized into two stages. Pain during the first stage of labor is mostly caused by the stimulation of the uterine chemoreceptors, which respond to stretching caused by uterine contractions (Labor and Maguire, 2008). The second stage of labor is associated with a more somatic component resulting from the stretching of the vagina and traction on the uterine ligaments and pelvic organs (Labor and Maguire, 2008). The characteristics of labor pain enable the observation of various changes in pain that occur over time within an individual, which can be useful for evaluating the performance of analgesic indices. Thus, we evaluated the performances of the SPI and ANI in conscious healthy volunteers and parturients.

## Materials and Methods

### Study Design and Procedure for Volunteers

The volunteer-based study incorporated a randomized, two-period, cross-over design. The study protocol was approved by the institutional review board of Asan Medical Center (approval number: 2014–0309) and registered with an international clinical research information system (^1^ KCT0001808, date of registration: February 11, 2016). All methods were performed in accordance with the relevant guidelines and regulations of the institution. Ten healthy young volunteers were enrolled after obtaining informed written consent. The exclusion criteria included autonomic nervous system disorders, arrhythmia, use of sedatives, history of neurosurgery, psychiatric diseases, epilepsy, pregnancy, and any neuromuscular disease evoking spontaneous pain.

**FIGURE 1:**
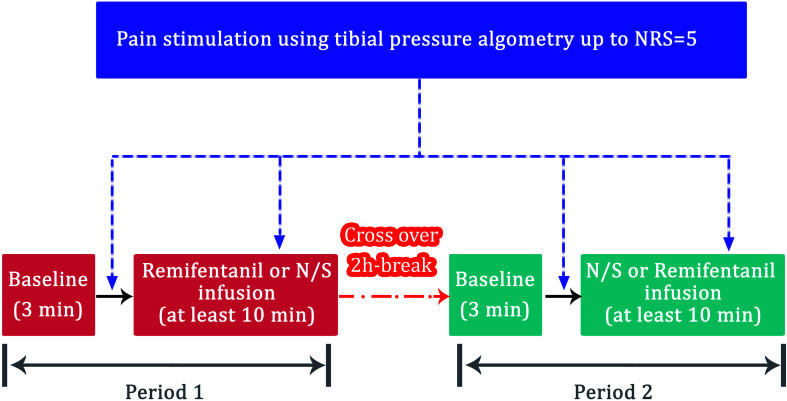
Flow diagram for the volunteer study (*n* = 10). NRS, numeric rating scale (0 = no pain; 10 = the most severe pain); N/S, normal saline.

All volunteers were monitored using electrocardiography (ECG) and pulse oximetry in the operating room. A reusable SPI sensor (Carescape^®^ B850; GE Healthcare, Milwaukee, WI, United States) was placed on the index finger of one arm. The ANI values were obtained using the ANI electrodes in V1 and V5 ECG positions and were continuously displayed on a stand-alone ANI monitor (MetroDoloris, Lille, France). The SPI and ANI values were recorded on a laptop for offline analysis. The study comprised two periods (Figure 1). The volunteers were allowed to acclimatize to the surroundings for at least 10 min in the supine position in a quiet operating room, after which the baseline data (without pain) were collected for 3 min. A painful algometric stimulus was applied with an algometer composed of a piston with a pressure area of 1 cm^2^, and control components were fixed over the anterior tibial bone 15 cm distal to the patella bone. The force driving the piston was manually increased by 25 or 30 N every 10 s until the volunteer rated their pain as five on the NRS. The pressure was maintained for 1 min after reaching an NRS score of 5. The volunteers were allowed to rest for 15 min after the first pain stimulation. The first painful algometric stimulus was applied without any infusion, and the second stimulus was applied at least 10 min after achieving a pseudo-steady state between the blood and brain during the infusion of normal saline or remifentanil. The administration time was set to at least 10 min based on the results of a previous study. The amount of remifentanil required to effectively suppress the noxious stimulus of endotracheal intubation was 135 μg (Park et al., 2018). The administration time of remifentanil was set to at least 10 min after accounting for the target effect-site concentration and the volunteer’s weight because the painful algometric stimulus was required after administration of at least this dose or more. The infusion of the target effect-site concentration (3 ng/mL) of remifentanil or normal saline was controlled using the Minto model (Asan Pump, version 2.1.3, Bionet Co., Ltd., Seoul, South Korea,^2^, last accessed: June 24, 2014) (Minto et al., 1997). The target concentration was set to 3 ng/mL was based on the results of our previous study. An effect-site concentration of remifentanil 9.11 ng/mL was associated with a 50% probability of occurrence of muscle rigidity (Choe et al., 2017). According to the results of the pharmacodynamic model constructed in the previous study (Choe et al., 2017), the target concentration was set to 3 ng/mL because muscle rigidity may occur when the concentration of remifentanil exceeds 4 ng/mL. The two study periods were separated by a washout interval of 2 h to avoid carryover effects. The same procedure was repeated once again, but the drug to be administered was changed. The volunteers were infused with different solutions in each period: normal saline in one period and remifentanil in the other in random order. Simple randomization was performed using a computer-generated allocation sequence. Volunteer randomization was conducted by a coordinator who was not involved in this study.

### Study Design and Procedure for Parturients

The protocol of parturient-based observational study was approved by the institutional review board of Asan Medical Center (approval number: 2014–0318) and registered at an international clinical research information system (see text footnote 1, KCT0001793, date of registration: February 01, 2016). All procedures were performed in accordance with the relevant guidelines and regulations of the institution. Ten women admitted for labor and delivery, who had elected to have natural childbirth were enrolled after obtaining informed written consent. The parturients were fully informed about the study protocols. Parturients with clinically significant cardiovascular, respiratory, or endocrine diseases, medications that could affect the heart rate or arrhythmia were excluded. Data collection for the ANI and SPI was performed in the labor ward prior to epidural catheter insertion. According to the standard of care, ECG monitoring was not performed during the data collection periods. SPI and ANI data were collected for 2 min when the parturient rated their pain as 0 (no pain), 5 (moderate pain), and 7 (severe pain) on the NRS, respectively.

### Statistical Analysis

This was not a confirmation study, and the sample size was not calculated owing to its exploratory nature. The sample size was also not calculated by other studies that evaluated the performance of SPI and ANI for this reason (Le Guen et al., 2012; Yan et al., 2017). The sample size was determined within the range in which the clinical trial was practically possible after considering various circumstances. Instead, we decided to assess the validity of the sample size by calculating the power based on the results. Statistical analysis was performed using SigmaStat 3.5 for Windows (Systat Software, Inc., Chicago, IL, United States) and GraphPad Prism 8.2.0 (GraphPad Software, Inc., La Jolla, CA, United States). The means of the SPI and ANI values obtained during baseline measurement in each volunteer were compared with the maximum and minimum values, respectively, obtained within 2 min after establishing the NRS 5 scores. The SPI and ANI data acquired during the three sequences in the volunteers [baseline (NRS 0), first pain stimulation (NRS 5), and second pain stimulation (NRS 5^‡^)] were compared using the one-way repeated measures ANOVA. The effect of these sequences was considered in the analyses. The mean values of SPI and ANI measured at NRS 0 in the parturients were compared with the maximum and minimum values, respectively, within 2 min after establishing the NRS 5 and NRS 7 scores. The SPI and ANI data obtained during the three sequences in the parturients (NRS 0, NRS 5, and NRS 7) were compared using the one-way repeated measures ANOVA. Data were expressed as mean ± standard deviation (SD) for normally distributed continuous variables, median (25–75%) for non-normally distributed continuous variables, and counts and percentages for categorical variables. *P*-values < 0.05 were considered statistically significant.

## Results

### Volunteers

**FIGURE 2:**
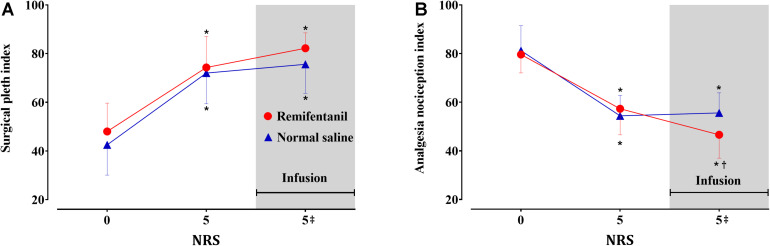
Surgical Pleth Index **(A)** and Analgesia Nociception Index **(B)** values in the volunteers (*n* = 10). Data are expressed as mean with the error bars representing standard deviation. Numerical rating scale for pain (NRS) 0: baseline, 5: first algometric stimulation in the absence of medication, 5^‡^: second algometric stimulation during infusion of remifentanil (red circle) or normal saline (blue triangle). Three sequences (NRS 0, 5, and 5^‡^) were compared using the one-way repeated measures analysis of variance (ANOVA) followed by a *post hoc* Holm–Sidak test. *P < 0.05 vs. baseline, ^†^*P* < 0.05 vs. NRS 5.

Sixty sets each of SPI and ANI measurements obtained from the 10 volunteers were analyzed. The volunteers’ characteristics are presented in Table 1. Algometric forces needed to induce bone pain and heart rate during the study periods are summarized in Table 2. The forces needed to obtain NRS 5 were higher during remifentanil infusion compared with those at the first algometric stimulation in the absence of any medication. The placebo effect was not observed during the normal saline infusion period. After algometric stimulation, the heart rates tended to be higher than those at baseline, and this increase in the heart rate was more prominent after the application of a greater algometric force during the remifentanil period (Table 2). The changes in the SPI and ANI values are presented in Figure 2. Significant differences were observed between the SPI and ANI values at NRS 0 and NRS 5, irrespective of the infusion of remifentanil or normal saline (*P* < 0.001). The SPI values were similar for NRS 5, irrespective of remifentanil administration, whereas the ANI values were lower for NRS 5 during remifentanil administration (*P* = 0.028).

**TABLE 1:** Characteristics of the study participants.

	Volunteers (*n* = 10)	Parturients (*n* = 10)
Male/female	7/3	0/10
Age, years	22.6 ± 1.5	30.7 ± 4.0
Height, cm	178.4 (167.6–179.8)	160.3 ± 4.1
Weight, kg	67.2 ± 10.7	66.7 ± 11.7
Gestational age, weeks	–	38.5 (38.0–39.0)
Mean infusion rate of remifentanil, μg/kg/min	0.19 ± 0.02	–

Data are expressed as the mean ± SD, median (25–75%), or count as appropriate. *The mean infusion rate was calculated as follows:

*$\mathrm{Mean}\,\mathrm{infusion}\,\mathrm{rate}=\frac{\mathrm{Total}\,\mathrm{dose}\,\mathrm{administered}\,\mathrm{during}\,\mathrm{study}\,\mathrm{period}}{\mathrm{Body}\,\mathrm{weight}\times \mathrm{infusion}\,\mathrm{duration}}.$*

**TABLE 2:** Algometric forces needed to induce bone pain and heart rate in the volunteers.

	Normal saline			Remifentanil		
	NRS 0	NRS 5	NRS 5*	NRS 0	NRS 5	NRS 5*
Algometric force, N	0	108.5 ± 55.0^†^	112.5 ± 53.0^†^	0	98.0 ± 41.0^†^	173.5 ± 55.4^†⁣‡^
HR, bpm	66.1 (59.8−71.6)	68.6 (58.7–71.5)	71.9 (60.9–75.2) ^†^	62.6 ± 7.3	65.7 ± 8.9	78.2 ± 17.0^†⁣‡^
RRI, ms	902.0 ± 104.3	895.2 ± 103.4	869.6 ± 87.7	963.3 ± 88.8	918.3 ± 98.9	784.9 ± 154.2^†⁣‡^

Data are expressed as the mean ± SD or median (25−75%) as appropriate. One volunteer (ID8) showed frequent premature atrial contraction on electrocardiography and was excluded from the HR and RRI analyses. NRS, numerical rating scale for pain; NRS 0, baseline; NRS 5, first algometric stimulation in the absence of any medication; NRS 5*, second algometric stimulation during infusion of normal saline or remifentanil; HR, heart rate; BPM, beats per minute; RRI, R-R intervals. Three sequences in the volunteers (NRS 0, NRS 5, and NRS 5*) were compared using the one-way repeated measures analysis of variance (ANOVA) followed by a *post hoc* Holm–Sidak test or Friedman repeated ANOVA by rank followed by a *post hoc* Tukey’s test. ^†^*P* < 0.05 vs. NRS 0, ^‡^*P* < 0.05 vs. NRS 5.

### Parturients

**FIGURE 3:**
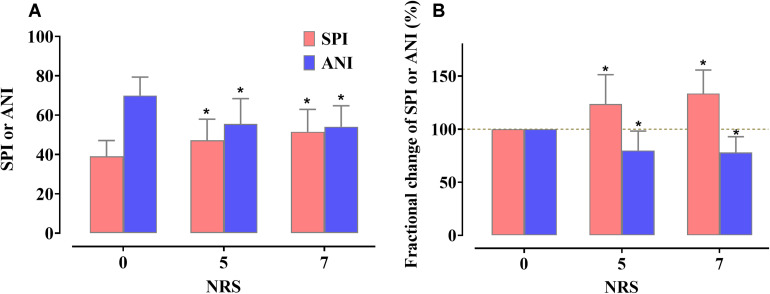
Actual value changes **(A)** and fractional changes **(B)** in the Surgical Pleth Index (SPI) and Analgesia Nociception Index (ANI) according to the change in the numerical rating scale (NRS) values. Data are expressed as the mean, with the error bars representing standard deviation. Three sequences (NRS 0, 5, and 7) were compared using the one-way repeated measures analysis of variance (ANOVA) followed by a *post hoc* Holm–Sidak test. *P < 0.05 vs. NRS 0.

A total of 16 parturients were enrolled in this study. Six parturients dropped out from the study due to withdrawal of consent (*n* = 1), failure to collect data due to childbirth (*n* = 3), and change to cesarean section (*n* = 2). Thirty sets of SPI and ANI data, each obtained from 10 parturients, were used for the analysis. The characteristics of the parturients are shown in Table 1. The changes in the SPI and ANI with respect to the NRS scores are shown in Figure 3. Both indices exhibited good distinction depending on the presence of pain (NRS 0 vs. NRS 5 or 7) but did not differ significantly between NRS 5 and NRS 7 (SPI: 47.2 ± 10.7 at NRS 5 and 51.5 ± 11.4 at NRS 7, *P* = 0.101; ANI: 55.5 ± 12.9 at NRS 5 and 54.1 ± 10.7 at NRS 7, *P* = 0.687).

## Discussion

The SPI and ANI differed significantly depending on the presence of pain as assessed by the NRS in both volunteers and parturients. The SPI values for NRS 5 were similar, irrespective of remifentanil administration at the target effect-site concentration of 3 μg/mL in volunteers, whereas the ANI values decreased with remifentanil administration. Neither index was able to distinguish between NRS 5 and 7 scores in parturients.

The results of our volunteer study showed that the SPI may be more appropriate for assessing pain under the influence of remifentanil from the perspective of its utility in accurately reflecting the degree of pain experienced by the patient. Pain is defined as an unpleasant sensory and emotional experience associated with actual or potential tissue damage or described in terms of such damage (Williams and Craig, 2016). Pain management is mainly based on the subjective evaluation of the patient’s complaint. The NRS for pain is mainly used in clinical practice, and an appropriate analgesic is administered based on the NRS score. For example, according to our hospital’s standards, surgical patients received tramadol 50 mg or pethidine 25 mg if their NRS score was more than four or seven, respectively (Jung et al., 2016). In other words, the NRS score determines the need for the type of rescue analgesic and its administration. Since the pain corresponding to the NRS score of five is inevitably different for each patient, an analgesic index that reflects the patient-determined NRS score is necessary in clinical settings. In the current volunteer study, the algometric force corresponding to five points on the NRS was higher when remifentanil was administered (Table 2), which can be interpreted as a logical result, because analgesics were administered in this situation. Pain can only be managed with the NRS score based on the patient’s response in actual clinical practice because it is impossible to know the actual intensity of pain corresponding to the algometric force. In fact, even if the pain is very severe, rescue analgesics are not administered unless the patient consent to it. Therefore, it is helpful for the quantitative analgesic index to be well-matched with the NRS score, irrespective of analgesic administration. Moreover, the ANI may have good responsiveness to opioids according to our results, although no study has evaluated the performance of opioid responsiveness for these indices to the best of our knowledge. Based on the results of this study, the ANI may be likely to overestimate pain in the presence of remifentanil administration. This difference between the two indices may be partly explained by the difference in their algorithms. The SPI value is determined by two variables, i.e., PPGA and HBI (Huiku et al., 2007). The weight of PPGA is approximately twice as great as that of the HBI. In contrast, the ANI value is based only on the HRV and is calculated as the surface of the filtered R-R interval obtained from ECG (Jeanne et al., 2009). Remifentanil has been shown to blunt the response of HRV to noxious stimuli (Luginbuhl et al., 2007), while the PPGA response was little affected by remifentanil (Struys et al., 2007). Hence, the SPI seems to be more precise than the ANI in reflecting pain, even with the infusion of remifentanil.

In the parturient study, neither index was able to successfully distinguish between moderate (NRS 5) and severe pain (NRS 7), which is in line with the results from previous studies: Choi et al. (2018) reported that SPI could not distinguish between moderate (3 ≤ NRS < 7) and severe (7 ≤ NRS < 10) postoperative pain, while ANI showed only a weak association with the NRS score in patients receiving sevoflurane during general anesthesia (Ledowski et al., 2013). However, other studies have reported that these indices could distinguish between varying degrees of pain (Le Guen et al., 2012; Boselli et al., 2013; Thee et al., 2015), with ANI showing a negative linear relationship with the visual analog scale in parturients (Le Guen et al., 2012). These conflicting results may be attributed in part to the difference in the study settings, including the type of anesthesia, opioid administration method, and pain assessment method. The type of sedative-hypnotic and opioid used during anesthesia may also affect these results (Boselli and Jeanne, 2014). Moreover, considering that studies with a larger sample size are better suited to distinguishing between pains of various intensities, the difference in the number of observations used for the analysis may have led to the difference in the results. Studies have suggested that nociception monitoring indices, such as the “traffic light scale,” may provide simpler information (Ledowski, 2019), which may be more meaningful than an inaccurate value between 0 and 100, when trying to determine the need for analgesic administration. From this perspective, the inability to distinguish between moderate and severe pain may not be critical drawbacks of SPI and ANI.

The HRV analysis may be an indicator of autonomic nervous system activity in response to pain stimulation. However, our study did not include an HRV analysis because the accuracy of HRV measurement was not guaranteed for the following reasons. First, the control of respiratory rate is important for accurate HRV analysis, especially high-frequency analysis (Bernardi et al., 2000); however, the respiratory rate could not be controlled in our study since it involved extreme conditions such as pain stimulation, thereby limiting the reliability of HRV. Moreover, in our study, data on baseline, pain stimuli, and drug infusion were obtained in 2-min sessions, which is shorter than the minimum 5-min measurement time recommended by the Taskforce of the European Society of Cardiology and Northern American Society of Pacing and Electrophysiology for HRV analysis (Camm et al., 1996). Previous studies have investigated whether ultra-short-term HRV (i.e., HRV obtained from ECG performed for less than 5 min) could be used as a substitute for the 5-min HRV; however, a recent review on ultra-short-term HRV studies suggested that the available studies have failed to provide a clear basis for validating ultra-short-term HRV (Pecchia et al., 2018), as the three studies that analyzed the effectiveness of ultra-short-term HRV have reported varying results (Esco and Flatt, 2014; Baek et al., 2015; Munoz et al., 2015). For example, the three studies suggested different time intervals of 10, 30, and 60 s, respectively, for the minimum time required for the analysis of the root mean square of the successive difference between the RR interval (RRI) (RMSSD) analysis, which cannot be accepted as generalizable results. Moreover, frequency domain or non-linear analysis are even more ill-equipped to verify the accuracy of ultra-short-term HRV. The most important limitation of using ultra-short-term HRV in our study lay in the experimental conditions. The pain stimuli or drug infusion performed in this study may be directly involved in autonomic nervous system activity, which is thought to be distinct from the resting state in HRV analysis. This also means that the results may be dependent on the length of the HRV analysis interval; thus, the application of ultra-short-term HRV must be confirmed for use in stimulus-application situations such as the ones used in the current study. However, all existing studies on ultra-short-term HRV have been performed in the resting condition and have failed to verify HRV under conditions of physical stress and drug infusion.

Our study had the following limitations. First, the number of observations in the volunteer group was relatively small compared to those used in previous patient-based studies. It is difficult to ignore the possibility of some form of distortion if the analysis is conducted with a small sample. According to the central limit theorem, replacing a given population with mean and SD with a sufficiently large random sample from the population results in a sample mean with an approximately normal distribution. If the sample size is large enough (typically *n* > 30), this theorem holds true whether the source population is normal or skewed (Kwak and Kim, 2017). The theorem holds true even for samples smaller than 30 if the population is normal. The distribution of SPI and ANI measured at each evaluation point passed the normality test for the volunteer and parturient investigations. The changes in the values of SPI and ANI induced by algometric forces were also consistent, and thus, the SD was not sufficiently large when considering the mean value (see Figure 2). The results of the power analysis for verifying the suitability of the sample size of the comparison groups based on the study’s results are summarized in the supplementary materials (Supplementary Table 1). Although the sample sizes were small, all comparisons between the groups that showed statistically significant results had powers of 0.85 or more. A future study involving a sufficiently larger study population would be useful to validate the results of the current study. Second, pain was assessed only at one point (NRS 5) in the volunteer study. A more detailed result using multiple pain intensity points would have been useful; however, it is unethical to cause pain beyond NRS 5 (moderate pain) in conscious healthy volunteers.

In conclusion, both the SPI and ANI were found to be effective in distinguishing the intensity of pain in healthy volunteers and parturients. The SPI showed similar values for perceived pain intensity, irrespective of remifentanil administration and would probably be more useful than the ANI to determine treatment based on pain assessment in clinical practice. Neither of these indices was able to distinguish between NRS 5 and 7 scores in parturients.

## Data Availability Statement

The raw data supporting the conclusions of this article will be made available by the authors, without undue reservation.

## Ethics Statement

The study protocol for volunteers was approved by the institutional review board of Asan Medical Center (approval number: 2014–0309) and registered at an international clinical research information system (http://cris.nih.go.kr, KCT0001808, date of registration: February 11, 2016). The patients/participants provided their written informed consent to participate in this study. The protocol of parturient-based observational study was approved by the institutional review board of Asan Medical Center (approval number: 2014–0318) and registered at an international clinical research information system (see text footnote 1, KCT0001793, date of registration: February 01, 2016).

## Author Contributions

J-YB, B-MC, and G-JN collected the data and designed the protocol. HS, J-HL, E-KL, and B-MC performed the data analysis. B-MC, HS, and G-JN interpreted the results. All authors contributed to the writing of the manuscript, provided critical revisions, and approved the final version.

## Conflict of Interest

The authors declare that the research was conducted in the absence of any commercial or financial relationships that could be construed as a potential conflict of interest.

## Footnotes

1 http://cris.nih.go.kr

2 http://www.fit4nm.org/download

## Supplementary Material

Supplementary Table 1 Power calculation to verify the suitability of the sample size of the comparison groups based on the study’s results.
