# Supplementary material for: Performance of the Surgical Pleth Index and Analgesia Nociception Index in Healthy Volunteers and Parturients
Source: Front Physiol. 2021 Mar 8;12:554026. doi: 10.3389/fphys.2021.554026 (PMC7982810; doi:10.3389/fphys.2021.554026)
Supplement: Supplementary Table 1 — Power calculation to verify the suitability of the sample size of the comparison groups based on the study’s results. [file Table_1.docx]

**Table S1**. Power calculation to verify the suitability of the sample size of the comparison groups based on the study’s results.

| Study | Analgesic index | Comparison group | P-value | Mean† | SD† | Power |
| --- | --- | --- | --- | --- | --- | --- |
| Volunteers | SPI | Remi 0 *vs.* Remi 5 | < 0.001 | − 26.3 | 10.2 | 1.0 |
|  |  | Remi 0 *vs.* Remi 5* | 0.002 | − 34.2 | 18.4 | 1.0 |
|  |  | Remi 5 *vs.* Remi 5* | 0.181 | − 7.9 | 17.3 | 0.42 |
|  |  | N/S 0 *vs.* N/S 5 | < 0.001 | − 29.5 | 17.0 | 0.99 |
|  |  | N/S 0 *vs.* N/S 5* | < 0.001 | − 33.1 | 15.3 | 1.0 |
|  |  | N/S 5 *vs.* N/S 5* | 0.205 | − 3.6 | 8.3 | 0.39 |
|  | ANI | Remi 0 *vs.* Remi 5 | < 0.001 | 22.3 | 16.1 | 0.99 |
|  |  | Remi 0 *vs.* Remi 5* | < 0.001 | 33 | 14.8 | 1.0 |
|  |  | Remi 5 *vs.* Remi 5* | 0.028 | 10.7 | 11.1 | 0.92 |
|  |  | N/S 0 *vs.* N/S 5 | < 0.001 | 26.8 | 13.0 | 1.0 |
|  |  | N/S 0 *vs.* N/S 5* | < 0.001 | 25.6 | 10.7 | 1.0 |
|  |  | N/S 5 *vs.* N/S 5* | 0.749 | − 1.2 | 11.5 | 0.09 |
| Parturients | SPI | NRS 0 *vs.* NRS 5 | 0.021 | − 8.2 | 9.3 | 0.87 |
|  |  | NRS 0 *vs.* NRS 7 | < 0.001 | − 12.5 | 7.5 | 0.99 |
|  | ANI | NRS 0 *vs.* NRS 5 | 0.007 | 14.5 | 13.1 | 0.97 |
|  |  | NRS 0 *vs.* NRS 7 | < 0.001 | 15.9 | 11.7 | 0.99 |

SPI: Surgical Pleth Index, ANI: Analgesia Nociception Index, Remi: remifentanil, N/S: normal saline, Remi 0 or N/S 0: baseline, Remi 5: first algometric stimulation without remifentanil infusion, Remi 5*: second algometric stimulation during infusion of remifentanil, N/S 5: first algometric stimulation without normal saline infusion, N/S 5*: second algometric stimulation during infusion of normal saline, †: mean or standard deviation (SD) of the difference between the two groups
